# Efficacy of dietary and lifestyle interventions in obesity management: a therapeutic protocol at the Diabetes Department, Marius Nasta Institute of Pneumophthisiology, Bucharest, Romania

**DOI:** 10.25122/jml-2024-0417

**Published:** 2025-03

**Authors:** Oana-Andreea Parlițeanu, Simona Carniciu, Alina Spinean, Cristiana Voineag, Beatrice Mahler

**Affiliations:** 1 Marius Nasta Institute of Pneumophthisiology Bucharest, Romania; 2 Carol Davila University of Medicine and Pharmacy, Bucharest, Romania; 3 Faculty of Medicine and Pharmacy, Dunarea de Jos University of Galati, Galati, Romania

**Keywords:** obesity, diet, lifestyle changes, weight loss

## Abstract

The primary objective of this study was to underline the importance of comprehensive medical education in the management of obesity. In clinical practice, patients frequently encounter challenges in achieving weight-related goals. Given that obesity is a major cause of global mortality, we aimed to evaluate the effectiveness of implementing targeted educational interventions on patient outcomes. To this end, we developed a specific dietary plan and educational materials for lifestyle modification administered to 44 patients in the Diabetes Department of Marius Nasta Institute of Pneumophthisiology in Bucharest, Romania. Assessments were conducted at baseline and after a three-month intervention period. The total body weight loss was 11%. BMI reductions were reflected in the redistribution of obesity types. The number of overweight individuals increased from 10 men (22.72%) to 13 men and 5 women, totaling 18 individuals (40.90%, with 29.54% men and 11.36% women). Blood glucose values dropped by 5%, and levels of HbA1c dropped by 0.4% from baseline to follow-up. The SAS severe group went down from 16 patients to 10 patients, and there was an increase in the mild Sleep Apnea Syndrome (SAS) group, increasing from 8 to 14, resulting in a 13.63% overall increase. Our findings indicate that enhanced engagement by the therapeutic team, combined with detailed educational resources and adequate time for their comprehension, improved patient health outcomes, led to weight reduction and a reduction in the severity of comorbidities such as hypertension, dyslipidemia, and sleep apnea, which translated to improved quality of life.

## INTRODUCTION

In 2022, the World Obesity Federation reported 746 million obese individuals globally [1]. Projections indicate that by 2030, this number will exceed 1 billion, affecting 1 in 5 women and 1 in 7 men. In Romania, adult obesity prevalence is 22.5% (ranging from 18.3% to 29% across different regions), making a number of approximately 4,320,000 individuals [2,3]. The 2023 edition of the World Atlas of Obesity predicts a concerning rise in Romania, with adult obesity expected to reach 35% by 2035, reflecting an annual growth rate of 2.1%. Additionally, childhood obesity is projected to increase by 5.6% between 2020 and 2035. Romania ranks 80th out of 183 countries evaluated for obesity prevalence [1].

Obesity has been recognized by the World Health Organization (WHO) as a chronic disease, marking the beginning of a management strategy to combat this growing global epidemic. This recognition has led us all to question whether humanity has any chance against this condition, and unfortunately, we are still searching for an answer. Recent epidemiological studies have shown that this epidemic currently affects over 2 billion people worldwide and continues to rise [4].

Among the risks associated with obesity, we mention insulin resistance and its progression to type 2 diabetes, non-alcoholic fatty liver disease, hypertension, and multiple cardiovascular complications. The list goes on, including various forms of cancer and sleep apnea syndrome [4,5].

The current goal is to find a strategy to prevent obesity and overweight. If effective prevention methods cannot be developed, efforts should focus on treating obesity as efficiently as possible and at least preventing its complications. The most frequently discussed strategy has been lifestyle changes, involving a healthier diet and increased physical activity to balance energy intake and expenditure. Dietary modification strategies appear to be the most effective but must be implemented from childhood to become a healthy lifestyle rather than a short-term restrictive diet [4,6-9].

Dietary interventions that have shown results are those backed by short-term and long-term effect evaluations. To prove an intervention’s effectiveness, it must be tested for sustainability among healthcare professionals, and its financial aspect—specifically its cost-effectiveness—must also be evaluated [10].

In patient-centered policies, primary care offices emphasize pre-existing eating habits and religious affiliations, which may influence dietary preferences, tastes, and restrictions. A summary of effective diets has been compiled and published, providing inspiration for our research. Most cited studies on potentially beneficial diets emphasize lifestyle optimization by reducing food intake, initially achieving energy balance and subsequently a negative energy balance (intake vs. expenditure), accompanied by in- creased physical activity within tolerated limits [10].

The primary aim of this study was to investigate how comprehensive nutritional education and lifestyle guidance impact the management of overweight and obesity. We emphasize that investing time in explaining dietary and exercise strategies to patients is valuable, as it ultimately empowers them to confidently address the challenges of this condition.

## MATERIAL AND METHODS

We selected 44 patients from the Diabetes Department of Marius Nasta Institute of Pneumophthisiology who visited our practice between March and July 2023 and conducted quantitative re- search. Patient selection was based on the presence or absence of obesity, with exclusion criteria including individuals with type 2 diabetes receiving treatment with glucagon-like peptide-1 (GLP-1) analogs or those planning to initiate such treatment for obesity. The number of patients was not predetermined, so we included all patients who met the criteria during the specified period, resulting in a sample of 44 participants. We focused on patients managed solely through diet and lifestyle interventions. Evaluations were conducted at baseline and after 3 months of treatment, including physical examinations, anthropometric assessments, and clinical and biological evaluations at both time points. Standard physical examination was performed at baseline and follow-up. The exact anthropometric measurements were collected at both points in time: weight measured in kilograms (kg), height measured in centimeters (cm), and body mass index (BMI) calculated and expressed in kilogram per square meter (kg/m2). Clinical assessments at both time points monitored the presence of arterial hypertension, dyslipidemia, degree of obesity, and the stage of sleep apnea syndrome (SAS). Biological evaluations measured parameters such as blood glucose, glycated hemoglobin (HbA1c), total cholesterol, low-density lipoprotein (LDL) cholesterol, and triglycerides at initial and follow-up visits. In addition, sleep apnea was evaluated using the apnea/hypopnea index (AHI) to stage SAS and assess changes over time. Nocturnal polysomnography was conducted to determine the AHI and provide a detailed staging of sleep apnea at both assessments. This observational prospective study did not include a control group receiving standard obesity care for comparison, which is a limitation of the study.

In our practice, we have developed educational materials for patients that cover hydration, macronutrient composition, meal planning, food diaries, and tracking methods. We provided specific guidance for dining out, traveling, portion control, stress eating, sugar consumption, and special occasions like holidays and birthdays. We discussed strategies for managing hunger cues, both so- cial and internal, and addressed time constraints, acknowledging that preparing healthy meals often requires more time than ordering or cooking pre-packaged foods. Additionally, we covered clever shopping techniques, label reading, and maintaining a healthy diet on a budget. We encouraged daily eating, sleeping, and exercise routines, emphasizing their long-term benefits for strength and metabolism. Furthermore, we focused on cultivating good habits and healthy practices while phasing out detrimental ones, with regular measurements of each patient's progress.

**Figure 1:**
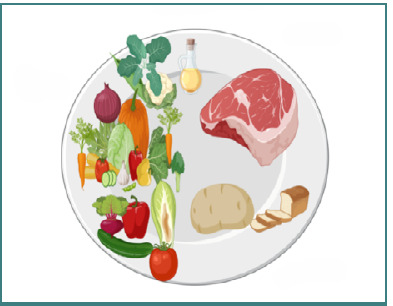
Ideal plate Created with BioRender.com

**Figure 2:**
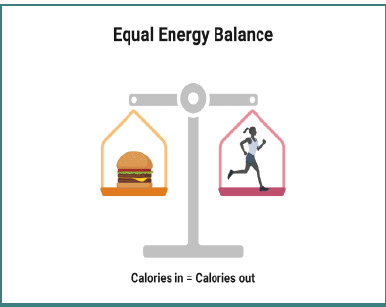
Equal energy balance Created with BioRender.com

**Figure 3:**
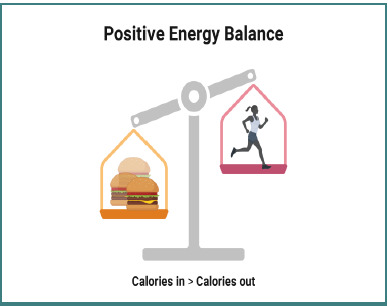
Positive energy balance Created with BioRender.com

We utilized graphic materials specifically created for our department using BioRender.com. Figures 1-3 were designed to convey our educational objectives, complemented by printed explanations. The images aimed to enhance patient education, adhering to the adage "an image is worth a thousand words". Patient feedback was positive, with many reporting that the visuals made the information more straightforward, easier to re- member, and more applicable at home. We used personalization techniques in our clinic to enhance patient training.

After evaluating each patient's initial nutritional status, we pro- vided them with these educational materials and a personalized nutrition meal plan tailored to their needs. Additionally, each patient received instructions regarding physical exercises, covering intensity, frequency, and duration. All parameters were reassessed and compared to baseline measurements during the follow-up examination. Patients were provided with a personalized dietary regimen that reduced overall caloric intake, with a targeted de- crease of 500-750 kcal per day on average. The dietary guide- lines emphasized a low-fat, low-carbohydrate approach, rich in protein and fiber throughout the day, alongside a recommended minimum water intake of 30ml/kg of body weight daily. Emphasis was placed on meals comprising substantial quantities of nutrient-dense foods while minimizing calorie consumption, such as salads, whole grains, fresh vegetables, and lean meats. The exercise plan advocated for a minimum of 30 minutes of physical activity every other day, summing at least 150 minutes per week, to establish a daily routine.

## RESULTS

A total of 44 patients were included in the study, with 29.55% (*n* = 13) being women and 70.45% (*n* = 31) men. Ages ranged from 40 to 77 years, with mean ages of 58.5 years for women and 55 years for men. All patients were from urban areas in Bucharest. Among them, 22.72% (*n* =10) were smokers, primarily males (18.18%, *n* = 8), and none reported alcohol or recreational drug use.

At baseline, men had a mean BMI of 43.0 kg/m^2^ (range 26.45–46.88 kg/m^2^), and women had a mean BMI of 42.92 kg/m^2^ (range 25.80–64.01 kg/m^2^). Type 1 Obesity (BMI 30–34.9 kg/m^2^) was present in 27.27% of patients, equally distributed between genders. Type 2 Obesity (BMI 35–39.9 kg/m^2^) affect- ed 25%, with a higher incidence in men (20.45%) than women (4.55%). Type 3 Obesity (BMI >40 kg/m^2^) was also present in 25% of the group of patients, equally distributed. Additionally, 22.72% were overweight (BMI 25–29.9 kg/m^2^), all men.

At baseline, 25% (*n* = 11) had arterial hypertension, 27.27% (*n* = 12) had dyslipidemia, 25% (*n* = 11) met the criteria for metabolic syndrome, 13.63% (*n* = 6) had impaired glucose levels or prediabetes, and 86.36% (*n* = 38) had sleep apnea syndrome. The initial mean systolic blood pressure was 155 mmHg for women and 165 mmHg for men, despite all patients being on antihypertensive medications according to guidelines.

Regarding the severity of SAS at baseline, 36.36% (*n* = 16) had severe SAS with an apnea/hypopnea index (IAP) over 30, predominantly men. Additionally, 31.81% (*n* = 14) had moderate SAS with an IAP between 15 and 29, and 18.18% (*n* = 8) had mild SAS with an IAP between 5 and 15.

Regarding lipid profiles, most patients were diagnosed with hypercholesterolemia, with an average total cholesterol of 262 mg/dL, an average LDL cholesterol of 143 mg/dL, and average triglycerides of 216 mg/dL. All patients previously diagnosed with dyslipidemia were on lipid-lowering agents (statins). Fur- thermore, 13.63% (*n* = 6) had baseline impaired glucose levels, with an average glucose value of 129 mg/dL and a mean HbA1c of 5.9% (Table 1).

**Table 1:** Mean measurements at baseline

Parameter	Total	Men	Women
Weight	114.5kg	107.5kg	121.5kg
Overweight	22.72% (*n* = 10)	22.72% (*n* = 10)	0
Type 1 obesity	27.27% (*n* = 12)	13.63% (*n* = 6)	13.63% (*n* = 6)
Type 2 obesity	25% (*n* = 11)	20.45% (*n* = 9)	4.55% (*n* = 2)
Type 3 obesity	25% (*n* = 11)	11.36% (*n* = 5)	9.09% (*n* = 4)
Arterial hypertension	25% (*n* = 11)	11.36% (*n* = 5)	9.09% (*n* = 4)
Dyslipidemia	27.27% (*n* = 12)	13.63% (*n* = 6)	13.63% (*n* = 6)
SAS severe	36.36% (*n* = 16)	22.72% (*n* = 10)	13.63% (*n* = 6)
SAS moderate	31.81% (*n* = 14)	18.18% (*n* = 8)	13.63% (*n* = 6)
SAS mild	18.18% (*n* = 8)	13.36% (*n* = 6)	4.55% (*n* = 2)
Systolic blood pressure		165mmHg	155mmHg
Total Cholesterol	262mg/dl	223mg/dl	289mg/dl
LDL	143mg/dl	112mg/dl	156mg/dl
Triglycerides	216mg/dl	189mg/dl	242mg/dl
Blood glucose	129mg/dl	128mg/dl	133mg/dl
HbA1c	5.9%	5.8%	6.1%

Patients were re-evaluated after 3 months of intensive nutrition- al treatment and lifestyle changes. The total body weight loss was 11%, decreasing from 114.5 kg to 101.91 kg across all patients. Men experienced a more significant loss of 13% (from 107.5 kg to 93.53 kg), while women lost 9% (121.5 kg to 110.57 kg).

BMI reductions were reflected in the redistribution of obesity types. The number of overweight individuals increased from 10 men (22.72%) to 13 men and 5 women, totaling 18 individuals (40.90%, with 29.54% men and 11.36% women). Type 1 obesity remained unchanged at 27.27% (*n* = 12), equally divided between men and women (13.63% each). In Type 2 obesity, the prevalence decreased from 25% to 22.72%, a reduction of 2.28%. The most significant change occurred in Type 3 obesity, which dropped from 25% at baseline to 9.09% at follow-up, a 15.91% reduction. Among men, it decreased from 11.36% to 2.27%, a 9.09% reduction; among women, it declined from 9.09% to 6.81%, a smaller reduction of 2.28%.

Although the proportion of patients with arterial hypertension remained at 25%, mean systolic blood pressure improved by about 15% in men and 10% in women. Dyslipidemia prevalence remained unchanged, but total cholesterol and LDL cholesterol dropped by 5%, and triglycerides fell by 20%. Blood glucose levels decreased by 5%, and mean HbA1c dropped by 0.4%, bringing all values below the diagnostic threshold for prediabetes (under 5.7% HbA1c and 126mg/dl for blood glucose).

In terms of sleep apnea severity, the percentage of patients with severe SAS decreased from 36.36% (*n* = 16) to 22.72% (*n* = 10). Men showed a reduction of 9.09% (from 22.72% to 13.63%), and women showed a reduction of 4.54% (from 13.63% to 9.09%). There was no change in the group with moderate SAS; the number remained the same, and so did the percentages. There was an increase in the mild SAS group, with the number of patients increasing from 8 to 14, resulting in a 13.63% overall increase, from 18.18% to 31.81%. Among men, the increase was 9.36%, from 13.36% to 22.72% (*n* = 10), and among women, it was 4.54%, from 4.55% to 9.09% (*n* = 4) (Table 2).

**Table 2:** Mean measurements at 3 months follow-up

Parameter	Total	Men	Women	Change	Change men	Change women
Weight	114.5kg	107.5kg	121.5kg	101.91kg (-11%)	93.53kg (-13%)	110.57kg (-9%)
Overweight	40.90% (*n* = 18)	29.54% (*n* = 13)	11.36% (*n* = 5)			
Type 1 obesity	27.27% (*n* = 12)	13.63% (*n* = 6)	13.63% (*n* = 6)	0	0	0
Type 2 obesity	22.72% (*n* = 10)	18.18% (*n* = 8)	4.55% (*n* = 2)	2.28%	2.27%	0
Type 3 obesity	9.09% (*n* = 4)	2.27% (*n* = 1)	6.81% (*n* = 3)	15.91%	9.09%	2.28%
Arterial hypertension	25% (*n* = 11)	11.36% (*n* = 5)	9.09% (*n* = 4)	0	0	0
Dyslipidemia	27.27% (*n* = 12)	13.63% (*n* = 6)	13.63% (*n* = 6)	0	0	0
SAS severe	22.72% (*n* = 10)	13.63% (*n* = 6)	9.09% (*n* = 4)	-13.64%	-9.09%	-4.54%
SAS moderate	31.81% (*n* = 14)	18.18% (*n* = 8)	13.63% (*n* = 6)	0	0	0
SAS mild	31.81% (*n* = 14)	22.72% (*n* = 10)	9.09% (*n* = 4)	+13.63%	+9.36%	+4.54%
Systolic blood pressure		165mmHg	155mmHg	140mmHg (-15%)	139mmHg (-10%)	
Total Cholesterol	262mg/dl	223mg/dl	289mg/dl	249mg/dl (-5%)	212mg/dl (-5%)	265mg/dl (-5%)
LDL	143mg/dl	112mg/dl	156mg/dl	136mg/dl (-5%)	106mg/dl (-5%)	148mg/dl (-5%)
Triglycerides	216mg/dl	189mg/dl	242mg/dl	173mg/dl (-20%)	151mg/dl (-20%)	193mg/dl (-20%)
Blood glucose	129mg/dl	128mg/dl	133mg/dl	122mg/dl (-5%)	121mg/dl (-5%)	115mg/dl (-5%)
HbA1c	5.5%	5.4%	5.7%	-0.4%	-0.4%	-0.4%

## DISCUSSION

The benefits of weight loss are well-documented, with a 10% reduction in body weight sufficient to reverse prediabetes [11]. In our study, an 11% weight loss among patients with impaired glucose or prediabetes correlated with a 5% decrease in mean blood glucose levels and a 0.4% reduction in HbA1c from base- line to follow-up. These results align with existing literature, as all patients achieved normal blood glucose and HbA1c levels by follow-up. A 5-15% reduction in body weight can positively impact arterial hypertension by lowering blood pressure values and reducing medication requirements [11]. Our patients experienced an 11% weight loss, decreasing systolic and diastolic blood pressure by 10% in women and 15% in men. However, this did not result in a reduction of antihypertensive medication; rather, it normalized blood pressure values under the same treatment.

Weight loss also has beneficial effects on SAS. Literature suggests that a 7–11% reduction in body mass can improve the apnea/hypopnea index (AHI) [11-13]. Our patients experienced an 11% weight loss, resulting in a shift from severe to milder forms of SAS at follow-up, consistent with literature findings.

Additionally, weight loss improves lipid profiles. A 5-15% reduction in body weight is known to lower triglycerides and LDL cholesterol while increasing HDL cholesterol [11,13-15]. In our study, an 11% weight loss correlated with a 5% decrease in LDL cholesterol and a 20% decrease in triglycerides. Research indicates that successful weight management requires detailed explanations, personalized meal plans, and a tailored approach for each patient's needs [16-19]. Our study and others highlight the necessity of extensive education, more time with patients, and individualized care strategies [20]. The management of obesity places a strong emphasis on dietary intervention. The diet must be high-quality, easy for patients to follow, understandable, and, perhaps most importantly, financially accessible. Additionally, such a diet should be accompanied by physical exercise—at least 150 minutes per week—to achieve positive weight loss outcomes [21].

In our study, we developed an affordable diet designed in a way that patients could easily understand, ensuring higher adherence in the long term. We combined this diet with physical exercises, maintaining the recommended 150 minutes weekly, with at least 30-minute sessions spaced no more than 2 days apart. Over the years, it has been demonstrated that a high-protein diet leads to greater weight loss compared to a low-protein diet. The best approach is to partially replace carbohydrates with low-fat protein sources, especially fast-absorbing carbohydrates with a high concentration of ultra-refined sugars [22,23].

Patients with obesity are, by definition, at a higher risk of developing obstructive sleep apnea syndrome and type 2 diabetes. A useful tool for detecting obstructive sleep apnea syndrome has been the STOP-BANG Questionnaire, particularly in patients with diabetes [24]. Although we have not used this questionnaire in our study, we aim to incorporate it in future research, along with the dietary and exercise tools we have developed in our clinic, to further refine the therapeutic protocol in the Diabetes Department of the Marius Nasta Institute. We believe that combining our materials with such a questionnaire will provide additional benefits to our patients, and we hope to identify other methods in the future to further improve our protocol.

The diet implemented in our study focused on increased in-take of protein and dietary fiber while reducing fats and carbohydrates. This nutritional approach facilitated weight loss and also contributed to improvements in sleep apnea symptoms, lipid profiles, and glycemic parameters.

A limitation of our study is the small sample size of 44 patients. This constraint prevented us from calculating statistical values such as *P* values or confidence intervals. Our approach to diet and lifestyle intervention is simple to use and can be easily scaled up if needed without demanding extensive training or long preparation periods for the medical professionals guiding patients. We believe this system can be effectively integrated into primary care facilities. While our results have shown positive outcomes in weight loss, enhanced quality of life, and better management of chronic diseases, we recognize that obesity is a chronic condition that requires ongoing effort for lasting results. We believe that consistently reminding our patients about healthy lifestyle choices, including diet and exercise, can lead to improved long-term outcomes.

## CONCLUSION

Prompt intervention with specially designed materials can improve patients' overall condition by reducing weight and thereby decreasing the high mortality risk associated with the chronic complications and comorbidities of obesity. By creating understandable, memorable, and easy-to-follow materials, we invest in patient care with the hope that these collaborative efforts between clinicians and patients will enhance the quality and longevity of life.
